# P-171. Epidemiology of Carbapenem-Resistant Gram-Negative Organisms across sites participating in CDC’s Global Action in Healthcare Network-Antimicrobial Resistance Module — Ethiopia, Greece, and India, October 2022-February 2024

**DOI:** 10.1093/ofid/ofae631.376

**Published:** 2025-01-29

**Authors:** Matthew Robinson

**Affiliations:** Johns Hopkins University School of Medicine, Baltimore, Maryland

## Abstract

**Background:**

A rising burden of carbapenem-resistant Enterobacterales (CRE) worldwide, in particular CRE producing plasmid-encoded carbapenemases (CPOs), has prompted global concern for rapid spread of resistance and difficult to treat infections. Data from low- and middle-income countries characterizing CRE is limited. CDC’s Global Action in Healthcare Network – Antimicrobial Resistance Module aims to prevent, detect, and respond to CP-CRE in healthcare settings.

**Figure:**
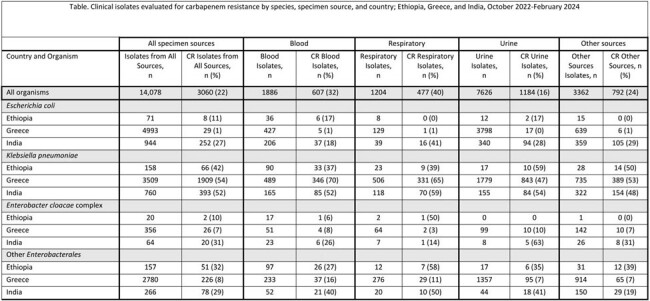

**Methods:**

CRE clinical isolates identified by antimicrobial susceptibility testing across select high-risk, inpatient, and outpatient settings in 8 hospitals in Ethiopia, Greece, and India were tested for carbapenemase production with modified carbapenem inactivation method and/or identification of KPC, NDM, VIM, IMP, and OXA-48-like carbapenemases using lateral flow assays (Greece and India) or KPC, NDM, VIM, and OXA-48-like using real-time polymerase chain reaction (Ethiopia).

Distribution of carbapenemase detection by country, Enterobacterales (N = 3,497) — Ethiopia, Greece, and India, October 2022-February 2024

**Figure:**
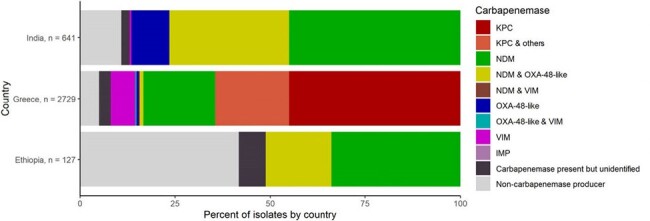

**Results:**

During October 2022– February 2024, 14,078 Enterobacterales isolates in 3 countries were evaluated for carbapenem resistance; 3,060 (22%) were carbapenem resistant (Table). Carbapenem resistance was common across settings for *Klebsiella pneumoniae* (42-54%) but was lower and more variable for *Escherichia coli* (1-27%) and *Enterobacter cloacae* complex (7-31%). Testing for carbapenemase production and/or mechanism was performed for 3,497 CRE isolates, which identified carbapenemase production or a mechanism in 3,239 (93%) of CRE isolates. Among CRE tested for carbapenemases, the most common carbapenemases identified were KPC in Greece and NDM in India and Ethiopia; multiple carbapenemases were identified in 793 (24%) of carbapenemase producers (Figure).

**Conclusion:**

In this global cohort, the majority of CRE tested for carbapenemase production were identified as carbapenemase-producing and the dominant carbapenemase detected by species varied by country. Collaboration, coordinated action, and innovative strategies are needed to both reduce spread of these highly resistant organisms in endemic settings and increase preparedness to prevent emergence and spread of novel carbapenem resistance mechanisms in the future.

**Disclosures:**

**All Authors**: No reported disclosures

